# Morphological Assessment of Breast Lesions With Type 2 Dynamic Curves Using DWI and T2WI Based on Breast Imaging Reporting and Data System Lexicon Descriptors

**DOI:** 10.1155/tbj/9957678

**Published:** 2025-09-05

**Authors:** Liying Zhang, Gongsheng Zhu, Kefan Wang, Tongzhen Zhang, Lin Lu, Xin Zhao

**Affiliations:** Department of Radiology, Third Affiliated Hospital of Zhengzhou University, Zhengzhou, China

**Keywords:** breast cancer, DWI, morphological assessment, T2WI, type 2 dynamic curves

## Abstract

**Purpose:** This study aimed to qualitatively assess the added diagnostic value of diffusion-weighted imaging (DWI) and T2-weighted imaging (T2WI), using Breast Imaging Reporting and Data System (BI-RADS) lexicon descriptors, in evaluating breast lesions with type 2 dynamic curves.

**Materials and Methods:** We retrospectively reviewed 181 breast lesions with type 2 dynamic curves in 181 consecutive patients who underwent 3-Tesla (3-T) magnetic resonance imaging (MRI). Trained radiologists assessed the morphological features of the lesions on dynamic contrast-enhanced (DCE) MRI, DWI, and T2WI using BI-RADS lexicon descriptors and measured the apparent diffusion coefficient (ADC). Statistical analysis was performed to compare variables in lesion type groups (mass-like group vs. nonmass-like group). Diagnostic performance was evaluated using the area under the receiver operating characteristic curve (AUC) and the DeLong test, with statistical significance at *p* < 0.05.

**Results:** In mass-like lesions, all morphological parameters significantly distinguished benign from malignant lesions on DCE, DWI, and T2WI (all *p* < 0.05). ADC values also showed significant differences (*p* < 0.05). The combined approach (DCE + DWI + T2WI) yielded the highest AUC (0.895), significantly outperforming the individual methods (all *p* < 0.05). In nonmass-like lesions, no parameter significantly predicted malignancy (all *p* > 0.05).

**Conclusion:** The addition of DWI and T2WI, interpreted using the BI-RADS lexicon descriptors, enhances the differential diagnosis of breast lesions with type 2 dynamic curves.

## 1. Introduction

Dynamic contrast-enhanced magnetic resonance imaging (DCE-MRI) is widely used for early breast cancer screening and diagnosis [1, 2]. DCE-MRI interpretation typically relies on morphological features and kinetic analyses in clinical practice [3, 4]. Time-intensity curve (TIC) analysis, obtained from the signal intensity (SI) values, is the most common method for kinetic MRI data. However, TIC analysis has certain limitations, as enhancement depends on various factors including contrast medium concentration, injected dose, the intrinsic characteristics of local vasculature, and imaging parameters (e.g., flip angle) [5, 6]. According to the 2013 Breast Imaging-Reporting and Data System (BI-RADS) MRI lexicon, TICs are classified into three general types. Among these, type 2 (plateau) dynamic curves often show considerable overlap between benign and malignant lesions. Plateau curves exhibit stable SI following peak enhancement, which typically occurs within 2–3 min. While these curves offer high sensitivity, they have relatively lower specificity in detecting malignant breast lesions [7, 8].

Unlike DCE-MRI, diffusion-weighted imaging (DWI) is a fast, functional, and noncontrast technique that shows promise in detecting and characterizing breast cancers [9, 10]. DWI analysis involves interpreting both raw DWI data and apparent diffusion coefficient (ADC) maps. ADC is a quantitative measure of water molecule diffusion in tissues. However, normal and pathological ADC values can vary depending on magnetic field strength, acquisition protocols, and measurement methods, which limit universal applicability [11–13]. According to the latest consensus from the EUSOBI International Breast DWI Working Group, the morphology, size, and location of lesions can be evaluated using DWI [14]. Additionally, previous studies have demonstrated that T2-weighted imaging (T2WI), which provides supporting information (available here), such as T2 hyperintensity within masses or perilesional edema, can improve diagnostic confidence in differentiating benign from malignant enhancements [15–18]. Wu et al. also reported that T2WI better delineates the morphological features of breast lesions [19].

Building on these findings, we introduced a systematized evaluation approach using BI-RADS lexicon descriptors to assess the added diagnostic value of DWI and T2WI, in comparison with DCE-MRI and ADC values, for distinguishing breast lesions with type 2 dynamic curves.

## 2. Materials and Methods

### 2.1. Patients

**Figure 1:**
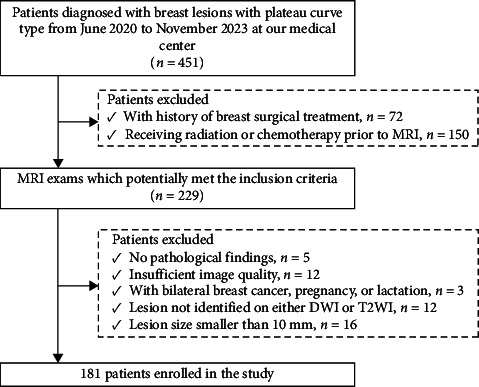
Flowchart of patient selection for our study.

Our Institutional Review Board approved this retrospective study and waived the requirement for informed consent. We retrospectively reviewed breast MRI examinations performed at our medical centers between June 2020 and November 2023 and included lesions with type 2 (plateau) dynamic enhancement curves. We excluded patients who had undergone prior breast surgical treatment or received neoadjuvant therapy before breast MRI. Additional exclusion criteria included the absence of pathological findings, poor image quality, bilateral breast cancer, pregnancy, lactation, lesion size < 10 mm, and lesions not visible on DWI or T2WI. Ultimately, 181 patients were enrolled in this study (Figure 1).

### 2.2. MRI

All breast magnetic resonance (MR) scans were acquired on a 3-T MRI scanner (Signa Pioneer, GE Healthcare, Milwaukee, WI, USA) using an 8-channel phased-array breast coil. Each patient was placed in a prone position and imaging was performed in the axial plane.

Precontrast imaging was obtained using three sequences: (1) a T2-weighted short tau inversion recovery images (repetition time [TR]/echo time [TE] = 7322/80.3 ms, echo train length = 24, field of view [FOV] = 320 × 320 mm^2^, matrix = 400 × 400, slice thickness = 5.0 mm, gap = 1.0 mm, and number of slices = 32); (2) the fast spin-echo T1-weighted images (TR/TE = 679/8.2 ms, echo train length = 4, FOV = 320 × 320 mm^2^, matrix = 455 × 400, slice thickness = 5.0 mm, gap = 1.0 mm, and number of slices = 32); and (3) the diffusion-weighted spin-echo echo planar imaging sequence with fat suppression (TR/TE = 3242.5/73.6 ms, FOV = 320 × 320 mm^2^, matrix = 128 × 128, slice thickness = 5.0 mm, gap = 1.0 mm, number of slices = 32, and *b* values = 0 and 800 s/mm^2^).

Two separate DCE-MRI datasets were acquired using ultrafast and standard DCE-MRI. Ultrafast DCE-MR images were acquired using a three-dimensional (3D) dual-echo fat-water-separated T1-weighted differential subsampling with Cartesian ordering sequence composed of a full k-space sampling phase, followed by 35 phases acquired continuously (TR/TE = 5.0/1.1 ms, FOV = 360 × 360 mm^2^, matrix = 300 × 164, thickness = 1.2 mm, temporal resolution = 7.4 s, and total scan time = 4 min 21 s). Standard DCE-MR images were acquired using a 3D fat-suppressed T1-weighted volume imaging breast assessment sequence composed of one precontrast and six postcontrast phases (TR/TE = 4.4/2.1 ms, FOV = 320 × 320 mm^2^, matrix = 320 × 320, thickness = 1.2 mm, temporal resolution = 74 s, and total scan time = 7 min 22 s). Dynamic imaging involved administering a body weight-adjusted dose of Gd-labeled diethylenetriaminepentaacetic acid (0.1 mmol/kg) intravenously at a rate of 2.0 mL/s to each patient, followed by 20 mL of saline flush at the same rate.

### 2.3. Image Analysis

All MR images were independently reviewed by two radiologists with 6 and 12 years of experience in breast MRI. Differences in opinion were resolved by consensus or consulting a third reader. Blinded to clinical and pathological details, radiologists visually confirmed the index lesion's curve type from MRI reports and subsequently analyzed the morphological characteristics of the lesions according to the fifth version of the BI-RADS lexicon descriptors. T2-weighted, diffusion-weighted, and DCE-MR images of all cases were evaluated on all lesion-containing slices; DCE-MRI was used as a reference to determine primary lesion areas for other images. Each lesion was classified by the type of enhancement as mass or nonmass type. In mixed lesions containing both mass and nonmass components, only the mass components were assessed. For patients with multiple lesions, only the largest mass on DCE-MRI was analyzed. Mass lesions were reported by shape (oval, round, or irregular), margin (circumscribed or not circumscribed), and internal patterns (homogeneous, heterogeneous, or rim), whereas nonmass-like lesions were reported by distribution (focal, regional, segmental, or diffuse) and internal patterns (homogeneous, heterogeneous, or clumped/clustered ring).

**Figure 2:**
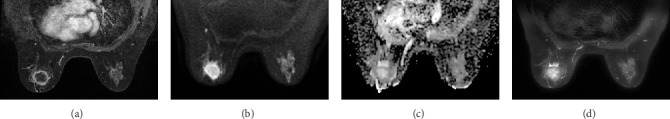
A 62-year-old female patient with metaplastic carcinoma in the left breast. (a) Dynamic contrast-enhanced image shows a round, uncircumscribed mass with rim enhancement in the left breast. (b–c) Diffusion-weighted image (DWI) acquired using a *b* value of 800 s/mm^2^ shows a round, circumscribed, and high-intensity mass. The internal pattern of combined assessment of DWI and apparent diffusion coefficient (ADC) was considered a rim-like pattern. The ADC value was 1.355 × 10^−3^ mm^2^/s. (d) T2-weighted image (T2WI) shows a high-intensity lesion surrounded by a low-intensity rim.

**Figure 3:**
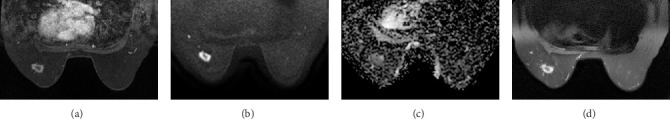
A 59-year-old female patient with invasive ductal carcinoma in the left breast. (a) Dynamic contrast-enhanced image shows an oval, circumscribed mass with rim enhancement in the left breast. (b–c) Diffusion-weighted image (DWI) acquired using a *b* value of 800 s/mm^2^ shows an oval, circumscribed, and high-intensity mass. The internal pattern of combined assessment of DWI and apparent diffusion coefficient (ADC) was considered a rim-like pattern. The ADC value was 0.825 × 10^−3^ mm^2^/s. (d) T2-weighted image (T2WI) reveals a low-intensity area surrounded by a high-intensity rim.

**Figure 4:**
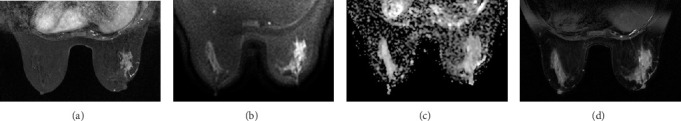
A 58-year-old female patient with ductal carcinoma in situ in the right breast. (a) Dynamic contrast-enhanced image reveals a regional nonmass lesion with homogeneous enhancement in the right breast. (b–c) Diffusion-weighted image (DWI) acquired by using a *b* value of 800 s/mm^2^ shows a regional high-intensity nonmass lesion extending in the anterior chest wall region. The internal pattern of combined assessment of DWI and apparent diffusion coefficient (ADC) was considered a heterogeneous pattern. The ADC value was 1.52 × 10^−3^ mm^2^/s. (d) T2-weighted image (T2WI) shows a regional, heterogeneous, and isointense nonmass lesion.

The internal enhancement pattern was substituted with internal signal characteristics suitable for analysis using DWI and T2WI, which are noncontrast sequences. SI on DWI and T2WI was recorded, when available, and categorized as low-iso, slightly high, or high relative to surrounding normal fibroglandular tissue. The internal patterns in DWI were described based on the most suspicious finding observed in high-*b*-value (*b* = 800) DWI images and ADC maps. Other morphological features were evaluated using high-*b*-value DWI alone. For T2WI, a higher or lower signal in the central portion of the lesion compared with the periphery was defined as a “rim sign,” with slight modifications according to previous studies [20, 21]. Typical examples of each type are shown in Figures 2, 3, and 4.

Radiologists analyzed the DCE and DWI images to localize the lesions and manually drew a region of interest (ROI) that encompassed the enhancing solid portion, corresponding to the area of lowest signal on the ADC maps, while excluding necrotic and hemorrhagic regions. ADC values were automatically calculated on the ADC maps, and the average of measurements by two independent readers was used for analysis. The largest transverse diameter of the index lesion was measured on DCE-MRI by consensus between the two readers.

### 2.4. Histopathological Analysis

All patients were pathologically confirmed using surgically resected or core needle biopsy specimens. Breast specimens were stained with hematoxylin and eosin and analyzed according to the World Health Organization classification of breast tumors. Histopathological data were obtained from the patients' original pathological reports.

### 2.5. Statistical Analysis

Statistical analyses were conducted using the R software, Version 4.1.0 (R Foundation for Statistical Computing, Vienna, Austria; https://www.R-project.org/). The R packages irr, dplyr, pROC, reportROC, and ggplot2 were utilized. The statistical significance level was set at *p* < 0.05. Lesions were stratified into mass and nonmass groups. Interobserver agreement in imaging findings between the two readers was evaluated using the intraclass correlation coefficient (ICC). Agreement was categorized as excellent (ICC > 0.9), good (ICC = 0.75–0.9), moderate (ICC = 0.5–0.75), or poor (ICC < 0.5). Fisher's exact test was applied to compare qualitative imaging characteristics between benign and malignant lesions. The Mann–Whitney *U* test was used to compare the quantitative ADC values. Logistic regression analysis was performed to assess the diagnostic utility of different methods in distinguishing benign from malignant lesions. The area under the receiver operating characteristic curve (AUC) was used to evaluate predictive performance. Differences between the AUCs were assessed using DeLong's test.

## 3. Results

### 3.1. Clinical Pathological Findings

All 181 patients were female. The mean age at diagnosis was 45.7 ± 12.3 years, and the mean lesion size was 31.9 ± 20.0 mm. Among the lesions, 94 (74.6%) mass-like and 32 (25.4%) nonmass-like lesions were malignant, whereas 45 (81.8%) mass-like and 10 (18.2%) nonmass-like lesions were benign. Clinical and pathological characteristics are listed in Table 1.

### 3.2. Interobserver Agreement

The ICCs for qualitative and quantitative imaging features exceeded 0.5, indicating moderate to excellent interobserver agreement. In mass-like lesions, ICCs were moderate to excellent for DCE (ICC = 0.844–0.886), DWI (ICC = 0.828–0.929), T2WI (ICC = 0.729–0.934), and ADC (ICC = 0.995). In nonmass-like lesions, the ICCs were also moderate to excellent: DCE (ICC = 0.851–0.986), DWI (ICC = 0.778–0.814), T2WI (ICC = 0.672–0.824), and ADC (ICC = 0.944). Full ICC results are listed in Supporting Table 1.

### 3.3. Qualitative and Quantitative Analyses

Qualitative imaging findings from the consensus readings were used for analysis. In mass-like lesions, significant differences in shape, margin, and internal patterns were observed between benign and malignant lesions on DCE, DWI, and T2WI (*p* < 0.05). Additionally, SI on DWI and T2WI significantly differed between benign and malignant mass-like lesions (*p* < 0.05). The mean ADC value was 1.486 ± 0.377 × 10^−3^ mm^2^/s for benign lesions and 1.088 ± 0.394 × 10^−3^ mm^2^/s for malignant lesions, indicating a statistically significant difference in the mass-like group (*p* < 0.05). There were no significant predictors of malignant breast lesions in the nonmass-like group (all *p* > 0.05) (Table 2).

### 3.4. Diagnostic Performance of the Methods

**Figure 5:**
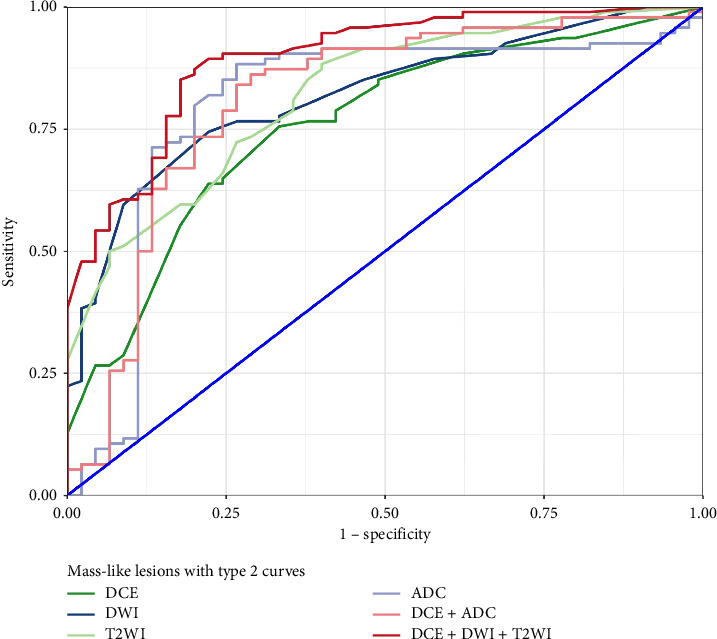
Receiver operating characteristic curves for various imaging methods in differentiating benign and malignant mass-like lesions with type 2 dynamic curves.

The diagnostic performances of various imaging methods in the differentiation of benign from malignant mass-like lesions with type 2 dynamic curves are presented in Table 3. For mass-like lesions, DCE, DWI, T2WI, and ADC demonstrated comparable diagnostic performance (AUC: 0.758 vs. 0.818 vs. 0.820 vs. 0.798, respectively, all *p* > 0.05). DeLong's test showed no significant difference between the DCE alone (AUC = 0.758) and DCE + ADC (AUC = 0.809) (*Z* = 1.837, *p*=0.066). The combination of DCE + DWI + T2WI yielded the highest accuracy (AUC = 0.895), significantly outperforming both DCE alone (*Z* = −3.776, *p* < 0.001) and DCE + ADC (*Z* = −2.27, *p*=0.023). Receiver operating characteristic (ROC) curves and corresponding AUC values for each method were plotted and compared (Figure 5 and Table 4).

## 4. Discussion

Dynamic enhancement curves aid in the clinical evaluation of breast lesions, particularly in distinguishing benign from malignant cases. Based on the shape of the enhancement curve, three general curve types are defined. Typically, benign lesions exhibit type 1 (persistent) curves, while malignant lesions more often display type 3 (washout) curves. Type 2 (plateau) curves, however, can occur in both benign and malignant lesions, posing diagnostic challenges [22]. In such cases, lesion morphology becomes the most important feature for assessment.

In this study, we focused specifically on breast lesions with type 2 dynamic curves and performed morphological analysis using DCE, DWI, and T2WI, along with quantitative ADC measurement. Lesions were stratified into mass-like and nonmass-like groups. Our results showed that the combination of DCE + DWI + T2WI achieved the highest diagnostic accuracy (AUC = 0.895), outperforming both DCE alone (*Z* = −3.776, *p* < 0.001) and the DCE + ADC (*Z* = −2.27, *p*=0.023) in differentiating benign from malignant mass-like breast lesions with type 2 dynamic curves.

ADC values can assist in differentiating malignant from benign lesions and have been associated with treatment response and prognosis in various malignancies [23, 24]. In our study, ADC was the only significant quantitative parameter for identifying malignant mass-like lesions with type 2 dynamic curves, consistent with findings by Ulu Ozturk et al. [7]. However, although the diagnostic efficacy of the DCE and ADC appeared similar, adding ADC did not significantly enhance predictive performance in the DCE + ADC combination. This may be attributed to our study's narrow focus on lesions with type 2 curves.

Previous studies have shown that qualitative morphological assessment of breast tumors on DWI using 3-T MRI is feasible [25–27]. However, limited research has explored the full range of BI-RADS descriptors in clinical DWI evaluation [11, 15, 28, 29]. Kul et al. reported a sensitivity of 92%–96% and specificity of 56%–67% for DWI-based mass assessment [11]. In contrast, our study showed a lower sensitivity (74.5%) but higher specificity (77.8%). This difference may result from our evaluation of internal patterns based on the most suspicious findings on both DWI and ADC maps. Additionally, our inclusion of iso- or low-signal mass-like lesions may have contributed to reduced sensitivity. For nonmass-like lesions, Kul et al. reported that the DWI signal was predictive of malignancy [11]; however, our results did not replicate this, likely due to our exclusive focus on type 2 dynamic curves.

Recent studies on T2WI have reported varied diagnostic utility depending on imaging features [19, 28, 30, 31]. In contrast to these, our study emphasized the use of BI-RADS lexicon descriptors on T2WI for evaluating type 2 curve lesions. DCE-MRI remains a reliable tool with high-resolution morphological visualization and strong sensitivity for detecting breast cancer [32]. Malignant lesions typically appear spiculated or irregular, with heterogeneous rim enhancement on MRI [33]. In our study, most malignant mass-like lesions with type 2 curves appeared ill defined (74.5%) and round or irregular (85.1%) on T2WI, findings consistent with DCE-MRI. Most breast cancers are hypointense on T2WI due to dense cellularity [33]. In our cohort, 54 of 94 (57.4%) malignant mass-like lesions with type 2 dynamic curves showed low or slightly high T2WI signal. Two ring-like T2WI patterns from prior literature were also observed [20, 21]. One pattern included the peripheral portion of the lesion, demonstrating a high signal and the central low signal, whereas the other showed central high SI with a peripheral ring of low SI on T2WI. Intratumoral high and low T2 SI is a characteristic finding of breast cancer, reflecting intratumoral necrosis and fibrosis, respectively [20, 21, 34, 35].

Baltzer et al. [36] reported that DWI + T2WI diagnostic performance is comparable to DCE-MRI. Similarly, we found that T2WI/DWI and DCE achieved equivalent accuracy when interpreted using BI-RADS descriptors. The combined use of all three modalities (DCE + DWI + T2WI) produced the best results (AUC of 0.895; *p* < 0.001), with sensitivity and specificity of 85.1% and 82.2%, respectively. The combination improved specificity without compromising sensitivity.

### 4.1. Limitations

Applying BI-RADS lexicon descriptors to evaluate the diagnostic performance of T2WI/DWI for breast lesions with type 2 dynamic curves has some limitations. First, the number of lesions analyzed, particularly nonmass-like enhancements, was relatively small. Therefore, our findings require validation in larger populations. Second, lesions that were not visible on T2WI or DWI were excluded from the analysis. Although most lesions in our study were detectable on T2WI/DWI, reduced visibility may increase the risk of false-negative results. With continued advances in MRI technology, such as improved fat suppression, distortion correction, and artifact reduction, higher resolution imaging may enhance the detection of subtle lesion features in the future.

## 5. Conclusion

Incorporating DWI and T2WI with DCE, based on BI-RADS lexicon descriptors, may improve the differential diagnosis of benign and malignant mass-like lesions with type 2 dynamic curves, particularly when diagnostic uncertainty exists, compared with using DCE and ADC alone. However, these findings should be considered preliminary, and validation through large-scale, multicenter randomized clinical trials is warranted.

## Figures and Tables

**Table 1:** Clinical-pathological characteristics of patients.

Variable	Malignant (*n* = 126)	Benign (*n* = 55)
Age (years)^a^	49.5 ± 10.8	36.8 ± 10.9
Lesion size (mm)^a^	33.6 ± 21.0	28.1 ± 17.1
Histological type		
IDC	92	
ILC	4	
Mucinous carcinoma	5	
Papillary carcinoma	4	
Apocrine carcinoma	1	
Metaplastic carcinomas	2	
DCIS	18	
Fibroadenoma		30
Fibrocystic changes		1
Papilloma		4
Sclerosing adenosis		3
Phyllodes tumors		3
Inflammatory changes		14

Abbreviations: DCIS, ductal carcinoma in situ; IDC, invasive ductal carcinoma; ILC, invasive lobular carcinoma.

^a^Data are mean values ± standard deviations. Unless otherwise specified, numbers in parentheses are percentages.

**Table 2:** Comparison of qualitative and quantitative findings obtained using the DCE, DWI, and T2WI.

	DCE		*p* value	DWI		*p* value	T2WI		*p* value	ADC		*p* value
	Benign	Malignant		Benign	Malignant		Benign	Malignant		Benign	Malignant	
Mass												
Shape												
Oval	18 (40.0)	12 (12.7)	0.001	19 (42.2)	18 (19.1)	0.012	18 (40.0)	14 (14.9)	0.005	1.486 ± 0.377	1.088 ± 0.394	< 0.001
Round	17 (37.8)	42 (44.7)		17 (37.8)	41 (43.6)		18 (40.0)	46 (48.9)				
Irregular	10 (22.2)	40 (42.6)		9 (20.0)	35 (37.2)		9 (20.0)	34 (36.2)				
Margin												
Circumscribed	26 (57.8)	27 (28.7)	0.001	29 (64.4)	38 (40.4)	0.011	26 (57.8)	24 (25.5)	< 0.001			
Not circumscribed	19 (42.2)	67 (71.3)		16 (35.6)	56 (59.6)		19 (42.2)	70 (74.5)				
Internal patterns												
Homogeneous	7 (15.6)	10 (10.6)	0.012	9 (20.0)	15 (16.0)	< 0.001	12 (26.7)	26 (27.7)	< 0.001			
Heterogeneous	32 (71.1)	49 (52.1)		34 (75.6)	44 (46.8)		33 (73.3)	48 (51.1)				
Rim	6 (13.3)	35 (37.2)		2 (4.4)	35 (37.2)		0 (0.0)	20 (21.3)				
Signal intensity												
Low ∼ Iso	—	—	—	2 (4.4)	0 (0)	0.010	9 (20.0)	41 (43.6)	0.021			
Slightly high	—	—		7 (15.6)	5 (5.3)		8 (17.8)	13 (13.8)				
High	—	—		36 (80.0)	89 (94.7)		28 (62.2)	40 (42.6)				
Nonmass												
Distribution												
Focal	2 (20.0)	4 (12.5)	0.672	2 (20.0)	4 (12.5)	0.634	2 (20.0)	4 (12.5)	0.634	1.223 ± 0.091	1.125 ± 0.258	0.260
Segmental	5 (50.0)	14 (43.8)		4 (40.0)	12 (37.5)		4 (40.0)	12 (37.5)				
Regional	3 (30.0)	8 (25.0)		4 (40.0)	10 (31.3)		4 (40.0)	10 (31.3)				
Diffuse	0 (0)	6 (18.8)		0 (0)	6 (18.8)		0 (0)	6 (18.8)				
Internal patterns												
Homogeneous	0 (0)	1 (3.1)	0.407	0 (0)	0 (0)	1.0	0 (0)	0 (0)	1.0			
Heterogeneous	6 (60.0)	25 (78.1)		9 (90.0)	29 (90.6)		10 (100.0)	31 (96.9)				
Clumped/clustered ring	4 (40.0)	6 (18.8)		1 (10.0)	3 (9.4)		0 (0)	1 (3.1)				
Signal intensity												
Low ∼ Iso	—	—	—	1 (10.0)	1 (3.1)	0.433	4 (40.0)	17 (53.1)	0.547			
Slightly high	—	—		3 (30.0)	7 (21.9)		5 (50.0)	9 (28.1)				
High	—	—		6 (60.0)	24 (75.0)		1 (10.0)	6 (18.8)				

**Table 3:** Diagnostic performance of the different methods in the diagnosis of benign and malignant mass-like lesions with type 2 dynamic curves.

	DCE	DWI	T2WI	ADC	DCE + ADC	DCE + DWI + T2WI
Sensitivity (%)	75.5	74.5	88.3	88.3	84.0	85.1
Specificity (%)	66.7	77.8	60.0	73.3	73.3	82.2
PPV (%)	82.6	87.5	82.2	87.4	86.8	90.9
NPV (%)	56.6	59.3	71.1	75.0	68.8	72.5
Accuracy (%)	72.7	75.5	79.1	83.5	80.6	84.2
AUC (95% CI)	0.758 (0.674∼0.842)	0.818 (0.748∼0.889)	0.820 (0.748∼0.891)	0.798 (0.707∼0.890)	0.809 (0.723∼0.896)	0.895 (0.840∼0.950)

Abbreviations: ADC, apparent diffusion coefficient; AUC, area under the curve; CI, confidence interval; DCE, dynamic contrast enhancement; DWI, diffusion-weighted imaging; NPV, negative predictive value; PPV, positive predictive value; T2WI, T2-weighted imaging.

**Table 4:** Comparison of *p* values for areas under the curve values of different methods evaluating benign and malignant mass-like lesions with type 2 dynamic curves.

Methods	DWI	T2WI	ADC	DCE + ADC	DCE + DWI + T2WI
	*Z*-value, *p* value	*Z*-value, *p* value	*Z*-value, *p* value	*Z*-value, *p* value	*Z*-value, *p* value
DCE	−1.537, 0.124	−1.875, 0.061	−0.922, 0.357	1.837, 0.066	3.776, < 0.001
DWI	N/A	−0.029, 0.977	0.401, 0.688	−0.211, 0.833	2.857, 0.004
T2WI		N/A	0.449, 0.653	−0.268, 0.788	2.847, 0.004
ADC			N/A	0.579, 0.562	2.209, 0.027
DCE + ADC				N/A	2.270, 0.023

Abbreviations: ADC, apparent diffusion coefficient; DCE, dynamic contrast enhancement; DWI, diffusion-weighted imaging; N/A, not applicable; T2WI, T2-weighted imaging.

## Data Availability

The data that support the findings of this study are available on request from the corresponding author. The data are not publicly available due to institution's policy.

## Supporting Information

A detailed description of the interclass agreement for qualitative parameters is offered in the Supporting Information.
