# Supplementary material for: Morphological Assessment of Breast Lesions With Type 2 Dynamic Curves Using DWI and T2WI Based on Breast Imaging Reporting and Data System Lexicon Descriptors
Source: Breast J. 2025 Sep 5;2025:9957678. doi: 10.1155/tbj/9957678 (PMC12431805; doi:10.1155/tbj/9957678)
Supplement: Supporting Information — Additional supporting information can be found online in the Supporting Information section. [file 9957678.f1.docx]

**Supplementary Table 1.** **Interclass correlation coefficient (ICC) for the parameters analyzed.**

|  | DCE | | DWI | | T2WI | | ADC | |
| --- | --- | --- | --- | --- | --- | --- | --- | --- |
|  | ICC (95% CI) | Level of concordance | ICC (95% CI) | Level of concordance | ICC (95% CI) | Level of concordance | ICC (95% CI) | Level of concordance |
| Mass |  |  |  |  |  |  |  |  |
| Shape | 0.886 (0.844～0.917) | Good | 0.927 (0.898～0.947) | Excellent | 0.934 (0.908～0.953) | Excellent | 0.995 (0.993～0.997) | Excellent |
| Margin | 0.881 (0.837～0.913) | Good | 0.828 (0.754～0.879) | Good | 0.729 (0.641～0.798) | Moderate |  |  |
| Internal patterns | 0.844 (0.789～0.886) | Good | 0.929 (0.902～0.949) | Excellent | 0.823 (0.761～0.870) | Good |  |  |
| Signal intensity | - |  | 0.899 (0.861～0.927) | Good | 0.925 (0.897～0.946) | Excellent |  |  |
| Non mass |  |  |  |  |  |  |  |  |
| Distribution | 0.986 (0.974～0.992) | Excellent | 0.784 (0.634～0.878) | Good | 0.771 (0.612～0.870) | Good | 0.944 (0.898～0.969) | Excellent |
| Internal patterns | 0.851 (0.737～0.917) | Good | 0.778 (0.625～0.874) | Good | 0.672 (0.467～0.809) | Moderate |  |  |
| Signal intensity | - |  | 0.814 (0.679～0.895) | Good | 0.824 (0.696～0.901) | Good |  |  |

DCE, dynamic contrast enhancement; DWI, diffusion-weighted imaging; T2WI, T2-weighted imaging; ADC, apparent diffusion coefficient; CI, confidence interval; ICC, intraclass correlation coefficient
